# Development of a national audit tool for juvenile idiopathic arthritis: a BSPAR project funded by the Health Care Quality Improvement Partnership

**DOI:** 10.1093/rheumatology/kex322

**Published:** 2017-10-23

**Authors:** Flora McErlane, Helen E Foster, Gillian Armitt, Kathryn Bailey, Joanna Cobb, Joyce E Davidson, Sharon Douglas, Andrew Fell, Mark Friswell, Clarissa Pilkington, Helen Strike, Nicola Smith, Wendy Thomson, Gavin Cleary

**Affiliations:** 1 Paediatric Rheumatology, Great North Children’s Hospital, Newcastle upon Tyne; 2 Institute of Cellular Medicine, Newcastle University, Newcastle upon Tyne; 3 Arthritis Research UK Centre for Epidemiology, Centre for Musculoskeletal Research, Institute for Inflammation and Repair, Faculty of Medical and Human Sciences, University of Manchester, Manchester; 4 Oxford Paediatric and Adolescent Rheumatology Centre, Oxford University Hospitals, Oxford; 5 Institute for Inflammation and Repair, Faculty of Medical and Human Sciences, University of Manchester, Arthritis Research UK Centre for Genetics and Genomics, Centre for Musculoskeletal Research, Manchester; 6 British Society for Paediatric and Adolescent Rheumatology Parents Group, London; 7 Scottish Network for Arthritis (SNAC), Glasgow; 8 Scottish Paediatric and Rheumatology Network (SPARN), Scotland; 9 Paediatric Rheumatology, Great Ormond Street Hospital, London; 10 Paediatric Rheumatology, Bristol Royal Hospital for Children, Upper Maudlin Street, Bristol; 11 Institute of Cellular Medicine, Newcastle University, Newcastle upon Tyne; 12 NIHR Manchester Musculoskeletal Biomedical Research Unit, Central Manchester NHS Foundation Trust, Manchester Academic Health Science Centre, Manchester; 13 Paediatric Rheumatology, Alder Hey Children’s Hospital, Liverpool, UK

**Keywords:** Juvenile idiopathic arthritis, outcomes, audit, patient reported outcome measure, patient reported experience measure, standards of care, quality

## Abstract

**Objective:**

Timely access to holistic multidisciplinary care is the core principle underpinning management of juvenile idiopathic arthritis (JIA). Data collected in national clinical audit programmes fundamentally aim to improve health outcomes of disease, ensuring clinical care is equitable, safe and patient-centred. The aim of this study was to develop a tool for national audit of JIA in the UK.

**Methods:**

A staged and consultative methodology was used across a broad group of relevant stakeholders to develop a national audit tool, with reference to pre-existing standards of care for JIA. The tool comprises key service delivery quality measures assessed against two aspects of impact, namely disease-related outcome measures and patient/carer reported outcome and experience measures.

**Results:**

Eleven service-related quality measures were identified, including those that map to current standards for commissioning of JIA clinical services in the UK. The three-variable Juvenile Arthritis Disease Activity Score and presence/absence of sacro-iliitis in patients with enthesitis-related arthritis were identified as the primary disease-related outcome measures, with presence/absence of uveitis a secondary outcome. Novel patient/carer reported outcomes and patient/carer reported experience measures were developed and face validity confirmed by relevant patient/carer groups.

**Conclusion:**

A tool for national audit of JIA has been developed with the aim of benchmarking current clinical practice and setting future standards and targets for improvement. Staged implementation of this national audit tool should facilitate investigation of variability in levels of care and drive quality improvement. This will require engagement from patients and carers, clinical teams and commissioners of JIA services.

Rheumatology key messages

- We report development of a national clinical audit tool for children with JIA.
- Staged implementation of national audit will inform future quality improvement interventions in JIA.
- We propose future development of an electronic data collection tool integrated into electronic patient records.

## Introduction

JIA is an umbrella term for a heterogeneous group of conditions characterized by chronic arthritis and categorized into sub-types according to clinical features at onset [1, 2]. JIA is one of the most common chronic inflammatory diseases of childhood with a UK incidence of 1:10 000 reported by Symmons *et al.* [3] in 1996. Although this equates to at least 1000 new cases per annum in the UK, the figure is almost certainly an underestimate; at that time, it was only possible to identify incident cases presenting to specialist paediatric centres. More recent incident data from other countries are higher [4, 5].

Appropriately tailored interventions are central to minimizing the adverse impact of JIA on physical, psychological and visual function, health-related quality of life and social/educational attainments [6]. However, there is wide variation in severity of presentation and disease course, and treatment-related morbidity can be significant. In part as a consequence of this heterogeneity, high quality evidence supporting best practice in JIA is limited, resulting in wide variations in service delivery.

The British Society for Paediatric and Adolescent Rheumatology (BSPAR)/Arthritis and Musculoskeletal Alliance Standards of Care (SOC) for Children and Young People (CYP) with JIA (2009) are consensus-derived minimum standards for clinical services delivering paediatric rheumatology (PRh) care [7]. The SOC were developed in accordance with national policy at the time [8, 9] and are endorsed by the Royal College of Paediatrics and Child Health. Key philosophies underpinning the standards included empowerment of patients and carers in treatment plans and a holistic approach to the provision of care. Despite the introduction of the SOC and an increasing awareness of the negative impact of delay in access to PRh care on disease outcomes in JIA [10], a 2013 multi-site UK audit against key SOC demonstrated considerable variation in service delivery and time to access specialist care [11]. In addition, the audit demonstrated a need for consensus agreed and measurable JIA-specific quality indicators, reflecting current clinical practice and including both clinician reported and patient/parent reported outcome measures, to enable standardization of clinical data collection. The 2013 audit formed the basis for a successful application to the Healthcare Quality Improvement Partnership (HQIP) to support development of the national audit tool for CYP with JIA. HQIP is an independent organization led by the Academy of Medical Royal Colleges, The Royal College of Nursing and National Voices and aims to promote quality in healthcare, in particular increasing the impact of clinical audit on healthcare quality improvement.

This paper describes the development of a nationally agreed tool and dataset for prospective national clinical audit of JIA. The audit tool will identify key service delivery measures necessary to benchmark current practice, and set future standards and targets for improvement; ultimately the aim is to reduce variations in delivery of care and improve health outcomes for CYP with JIA. To address the outcomes-based commissioning of National Health Service (NHS) services, a national clinical audit tool for JIA must include the UK NHS Specialist Commissioning for PRh specifications (NHS England Specialised Services Quality Dashboards) and also patient and carer reported outcome measures (PROMs) and patient and carer reported experience measures (PREMs).

In parallel to the development of this national audit tool for JIA, the UK PRh community has derived a standardized JIA-specific core dataset called Consensus derived, Accessible (information), Patient-focused, Team-focused, Universally collected (UK), Relevant to all and containing Essential data items (CAPTURE-JIA), to facilitate comparative clinical studies and research collaborations between providers of care [12]. The ultimate intention of the BSPAR community is to collect and embed the nationally agreed audit data items within CAPTURE-JIA, thereby incorporating indicators of service delivery, clinical outcomes, carer outcomes and experience into one dataset to be used in routine clinical practice to improve patient care.

The aim was to develop a national clinical audit tool for JIA that would enable evaluation of current clinical practice against both clinical outcomes and PROM/PREM. A secondary goal was to set future quality standards and to ensure these could be regularly assessed by standardized data collection with CAPTURE-JIA.

## Methods

### Development of governance structure

**Fig. 1:**
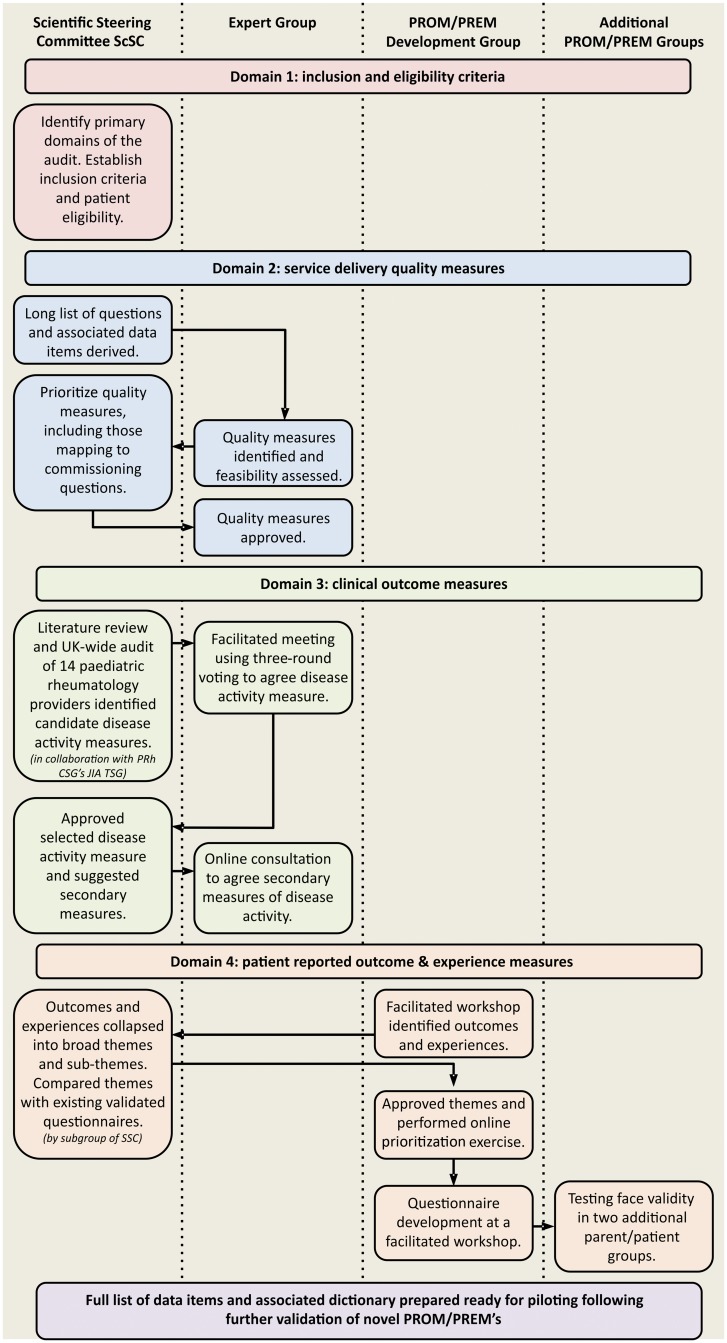
Methodology flowchart

To ensure engagement with all relevant stakeholders (clinicians, specialist nurses, allied health professionals, academics and parents/patients), a robust governance structure comprising three expert groups was established as follows. A complex and consultative methodology underpins the development of this JIA audit tool (Fig. 1).

#### Multidisciplinary scientific steering committee (ScSC) (n = 14 including representation from all stakeholders)

The committee was responsible for overall operational management and final decision making. Membership was open to individuals engaged with development of BSPAR SOC, CAPTURE-JIA and/or HQIP application.

#### Multidisciplinary expert group (n = 21, including 11 members of the ScSC with representation of all stakeholders)

The group provided expert guidance with powers to recommend actions. The expert group invited expressions of interest from all NHS England PRh providers (identified through the NHS Providers List) and PRh representatives from Scotland and Wales. A purposeful sample of respondents was invited to participate in the study, with >80% of all UK sites represented. The sample included a range of service delivery models and geographic locations, a spectrum of track records of research from the National Institute for Health Research (and other research involvement), PRh national training centres (termed ‘grid centres’) and other paediatric rheumatology centres (termed ‘non-grid’).

#### PROM/PREM development group (n = 17)

This group was responsible for development of novel PROM and PREM questionnaires suitable for national audit. The BSPAR National Parents Group was established in 2013 to identify and represent the experiences of families affected by musculoskeletal diagnoses, most commonly JIA. The PROM/PREM development group included the BSPAR Parents Group (with representatives from across England and Scotland), invited consumer representatives from the National Institute for Health Research, Clinical Research Network Children and Arthritis Research UK Clinical Studies Group and young people with JIA.

### Domains of audit

The ScSC established four domains for the audit, namely inclusion criteria and patient eligibility, service delivery quality measures and two aspects of impact, physician reported outcome measures and PROM/PREMs.

### Inclusion criteria and patient eligibility

The ScSC agreed inclusion criteria and eligibility. Discussion focused on the time window for collection of audit data, follow-up time per patient and decision to include only new patients or all patients attending rheumatology outpatients within a given audit time frame. Advice was taken from the Chair of the British Society for Rheumatology (BSR) Clinical Affairs Committee, which is responsible for the National Clinical Audit for Rheumatoid and Early Inflammatory Arthritis (BSR EIA) [13].

### Quality measures

An iterative approach between the ScSC and expert group was taken. The ScSC derived a preliminary long list of possible questions addressing key quality measures based on the BSPAR Standards of Care, the Scottish Paediatric and Adolescent Rheumatology Network Quality Standards, clinical commissioning requirements in England (NHS England dashboard for PRh) and additional suggestions from the wider ScSC. The specific data items required for each quality measure were listed and duplicate data items were removed to form a single list.

The feasibility of collecting the proposed list of data items was discussed with the expert group, highlighting the need for further prioritization. The ScSC prioritized the quality measures on the basis of perceived importance, inclusion in the commissioning dashboard and audibility (i.e. ease of collection of the necessary data items or overlap with CAPTURE-JIA). The expert group were asked to approve the final list.

To understand the impact of variation in service delivery, the quality measures will need to be assessed against the most important clinician reported outcomes and PROMs/PREMs.

### Clinical outcome measures

The ScSC agreed that the primary outcome measure would be a measure of disease activity and worked in consultation with the Clinical Studies Group JIA Topic Specific Group to develop a list of candidate disease activity measures informed by a literature review of disease activity assessment tools [14] and an audit of disease activity assessment in clinical practice [12]. All recognized PRh NHS England providers and representatives from Scotland and Wales were invited to participate in the audit.

At a facilitated meeting of the expert group, a three-round nominal group voting exercise was used to achieve consensus (majority agreement of ⩾70%) on the primary disease activity measure for inclusion. A consensus definition of 70% was selected to align our work with consensus definitions favoured by the OMERACT collaboration [15] and the Core Outcome Measures in Effectiveness trials initiative [16]; the consensus cut-off of 70% has been used in a number of previous paediatric rheumatology studies [17–19]. Following approval by the ScSC, secondary measures were agreed via an online consultation process with the expert group.

### PROMs/PREMs

Inclusion of PROMs and PREMs was a core requirement of the project. The PROM/PREM development group led the development and selection process, using a staged approach.

Patients and parents attended a facilitated workshop in which key outcomes and experiences for CYP with JIA were identified. The open exchange workshop was moderated by a PRh Consultant (N.W.). Key messages were brought together during and after the workshop by one of the consumer members of the BSPAR Parents Group (S.D.).

#### Identification of broad themes and sub-themes

Similar items were collapsed into a single item and grouped into broad themes and sub-themes by a sub-group of the ScSC. These were approved by the BSPAR Parents Group.

### Comparison with existing validated questionnaires

Any pre-existing validated questionnaires capturing any of the broad themes or sub-themes prioritized by the patient/parent group were considered for the audit. Factors considered included length of questionnaire, ease of scoring and the time referent period used.

#### Online prioritization of broad themes and sub-themes

A simple Yes/No online consultation exercise was undertaken with all members of the PROM/PREM development group to determine whether they would prefer one broad question capturing several aspects of a theme, or one specific question addressing just one sub-theme.

#### Questionnaire development workshop

A second facilitated workshop was held to finalize the development of the PROMs and PREMs. The goals for this final workshop were to agree the number of patient outcome and patient experience questions for inclusion, agree the time reference period for questions (e.g. today, in the last week, in the last month) and develop wording for the questions and the format for the answers, ensuring that the questions were (i) generic (i.e. transferrable to other paediatric musculoskeletal conditions), (ii) relevant and valuable to all patients/parents and (iii) suitable for inclusion in CAPTURE-JIA to facilitate clinical adoption.

Following the meeting, a further online exercise with all members of the patient/parent group was undertaken for refinement and final approval of the HQIP audit PROM/PREM questionnaires.

#### Testing face validity of the questionnaire

Two additional groups of parents attending one of two JIA family days in April 2016 (group 1: 20 parents attending a Scottish Network for Arthritis for Children weekend in Scotland; group 2: 11 parents attending a JIA family day in Oxford) were asked to complete the PROM/PREM questionnaires and an additional brief questionnaire including questions on completion time, ease of completion and ease of understanding. A group discussion to highlight any key issues was facilitated by one of the research team. 

A full list of data items and an associated data dictionary were prepared.

## Results

Membership of each of the three expert groups is provided online (supplementary Table S1, available at *Rheumatology* Online).

### Inclusion criteria and eligibility

JIA is an uncommon disease with relatively low numbers of new patients presenting year-on-year. Therefore, to ensure capture of sufficient data for meaningful analysis within a reasonable audit time frame, the ScSC agreed that all JIA patients (new and follow-up) be included.

### Quality measures

The ScSC identified 14 potential quality measures requiring the collection of 40 data items. Overall the expert group felt that the data required for this would be too extensive in the absence of an electronic data capture system. The ScSC therefore agreed to prioritize 11 quality measures requiring 32 data items, including all those that map to the commissioning questions (Table 1). The expert group approved the final list.

**Table 1:** Prioritized quality measures

Subject area		Proposed question
1. Categorization	1A	What is the number of patients in each ILAR sub-group in the audit population?
	1B	What is the proportion of patients in each ILAR sub-group, relative to the audit population?
2. Access	2	What is the median time for children with suspected JIA from receipt of the referral letter in the rheumatology department to the date of the first appointment offered in a rheumatology clinic? (modified PRH03)
		*(PRH03: children with newly diagnosed JIA should have access to a specialist paediatric rheumatology service within 6 weeks of the referral being received by the specialist service)*
3. Steroids	3A	What is the mean number of days to joint injection on a dedicated paediatric general anaesthesia list from date of decision to treat, for children of different ILAR sub-types? (PRH04)
		*(PRH04: Children with JIA who need to have intra-articular steroid injection(s) should wait no longer than 4 weeks for the procedure. Those needing general anaesthesia will have these performed on a paediatric general anaesthesia list)*
	3B	What percentage of children of different ILAR sub-types is on oral (systemic) steroids at different times after their first rheumatology clinic visit?
4. DMARDS	4	What is the median time from their first clinic visit to the decision to treat with methotrexate, for children of different ILAR sub-types?
5. Biologic therapies	5	What is the median time from their first clinic visit to the decision to treat with their first biologic therapy?
		– for children of different ILAR sub-types
		– for different biologic therapies
6 Uveitis	6	What is the median time from the patient's first clinic visit to the date of their first uveitis screening with an appropriate paediatric ophthalmic specialist, for patients of different ILAR sub-types? (modified PRH05)
		*(PRH05: Children with JIA should have access to uveitis screening within 6 weeks of diagnosis)*
7. Clinic organization	7A	What proportion of children who started a DMARD or biologic agent were counselled by a paediatric rheumatology clinical nurse specialist (PRH01)
		*(PRH01: Children with established rheumatic diseases (and their carers) should be counselled by a paediatric rheumatology clinical nurse specialist before starting treatment with a DMARD or Biologic)*
	7B	What proportion of children with JIA is seen in a specialist paediatric rheumatology clinic and what proportions in other clinic types (modified PRH02)
		*(PRH02: Children with JIA should have access to a paediatric rheumatology clinic for follow-up appointments)*
8. Research	8	What proportion of eligible patients has been recruited to the BSPAR Cohort Studies (BSPAR Etanercept and BCRD)?

Where a measure maps to a commissioning item then the commissioning statement is also shown in italics.

### Clinical outcome measures

A literature review informed the identification of 19 candidate disease activity measures (12 single and 7 composite measures) by the ScSC in collaboration with the JIA Topic Specific Group. A subsequent audit of current practice was completed by 10/14 invited PRh providers (71%), with data available for 153 children (median age 12 years, range 1–21 years). Although there was widespread agreement around important single measures of disease activity (100% centres intended to collect the core outcome variables, pain and uveitis), collection and documentation of these measures was inconsistent and unreliable, ranging from 87% (active joint count) to 43% (presence/absence of active uveitis) [12].

This information informed the expert group workshop. Although some members of the expert group had completed the audit, the expert group included a wider spectrum of centres and non-clinician stakeholders. The expert group achieved consensus (>70% agreement) on the three- or four-variable Juvenile Arthritis DAS as the primary outcome measure [20, 21] and presence/absence of uveitis (Y or N) as a secondary outcome.

The ScSC approved the choice of outcome measures but following some discussion, a simple yes/no consultation process with the expert group led to two modifications; documentation of presence/absence of sacroiliitis in enthesitis related arthritis and inclusion of the systemic Juvenile Arthritis DAS composite measure when published. The final list of clinical outcome measures is given in Table 2.

**Table 2:** Measures of clinical outcome, patient reported outcome and patient experience for assessment against quality measures

Outcome or experience		Proposed measure
Clinical measures of outcomes	1A	For each ILAR sub-group, what is the median JADAS3 score at different times from the first clinic appointment?
	1B	What proportion of patients of different ILAR sub-types have uveitis
	1C	What proportion of patients with enthesitis related arthritis have sacroiliitis
Patient reported outcome measures	2A	For each ILAR subtype, what proportion of children (categorized as never, sometimes, often or most of the time) experience interference with their daily activities due to: fatigue, pain, poor sleep, medication (side effects)?
	2B	For each ILAR sub-type, what proportion of children (categorized as never, sometimes, often or most of the time) have experienced interference with their ability to participate in the things they like to do (e.g. playing sport, going to the park, playing out, socializing with friends) due to their condition?
	2C	For each ILAR sub-type, what proportion of children (categorized as never, sometimes, often or most of the time) have felt sad/worried or frustrated?
Patient reported experience measures	3A	What proportion of patients/families felt that their questions and concerns had been answered in a way that they understood (categorized as not at all, a bit, mostly, fully)?
	3B	What proportion of patients/families understand their treatment plan (categorised as not at all, a bit, mostly, fully)?
	3C	What proportion of children/families feel supported in between visits (categorized as not at all, a bit, mostly, fully)?
	3D	What proportion of children/families felt that the environment in which they waited was suitable for those attending the appointment (categorized as not at all, a bit, mostly, fully)?
	3E	What proportion of patients experienced an unacceptable delay (categorized as no unacceptable delay, <15 min, 15–30 min, 30 min to 1 h, 1–2 h, >2 h)?

JADAS3: 3 variable Juvenile Arthritis Disease Activity Score.

### PROMs and PREMs

The initial PROM/PREM development group workshop initially identified 40 outcomes and 63 experiences. The outcomes and experiences were subsequently collapsed into three broad themes with 13 sub-themes for PROMs and six broad themes with 17 sub-themes for PREMs (Table 3).

**Table 3:** Patient/carer reported outcome and experience measure themes and sub-themes

PROM		PREM	
Main theme	Sub-theme	Main theme	Sub-theme
Physical well-being	Medication and treatment Physical effects Pain Fatigue/sleep	Communication	Consistency of advice/care Relationship with team Empathy and respect from staff
		Information/education	Ease of understanding Education Financial advice
Social well-being	Family impact Education effects Lifestyle	Access/coordination of care	Journey to diagnosis Access to all disciplines as required Access to treatments Ease of contact with health care professional Appointment times
		Needs/involvement	Emotional support Involving young person in their healthcare Transition experience
Emotional well-being	Uncertainty Confidence Frustration Support Invisibility Transition	Environment	Hospital/clinic environment Waiting room environment Convenience of hospital location Travel burden
		Confidence	Confidence/trust in medical team Confidence/trust in treatment plan

PREM, patient and carer reported experience measure; PROM, patient and carer reported outcome measure.

For PROMs, some existing questionnaires were identified that captured many of the same themes, for example, Child Health Utility 9D Index (CHU-9D) [22]. However, these were all considered too long or the scoring system too complicated for use in an audit. In addition, the referent time point form most questionnaires was today only; the group considered this too short a time frame to provide useful information.

For PREMs, just two pre-existing tools were identified: The Friends and Family questionnaire [23] and the PREM developed as part of the BSR EIA [24]. The Friends and Family questionnaire was considered to be too generic. The adult inflammatory arthritis PREM has many overlapping domains and could potentially have been adapted; however, once again the group considered this to be too long.

A prioritization exercise was sent to the PROM/PREM development group to identify broad themes and sub-themes for inclusion and there was an 88% (15 people) response rate. At the start of the second workshop, those attending (n = 8) agreed a cut-off of 11/15 (73%) to be the minimum requirement for inclusion of each theme in the audit. On initial review, no single broad theme or sub-theme met these criteria. Discussion continued and the group achieved consensus (>73%) within the meeting, enabling development of appropriate question and response options for each theme. Once finalized, the questions were sent to the wider parent/young person group (initial 15 respondents), and consensus was once again achieved.

Given that JIA is a relapsing and remitting condition that can vary widely over relatively short time periods, a referent period of 1 month was agreed as sufficient to capture variability but short enough to minimize recall bias.

Following the workshop, the final set of questions was sent to all members of the PROM/PREM development group and minor modifications made based on feedback. The final PROM and PREM against which the quality measures will be assessed are shown in Table 2. The PROM/PREM questionnaires are shown in Table 4.

**Table 4:** Parent/carer version of PROM and PREM questionnaires

Parent/carer version of PROM and PREM questionnaires (children aged <11 years)								
Completed by:	Mother		Father		Other (relationship to child)			
	□		□		□			
PROM: To be completed prior to seeing the doctor								
Patient outcome	Never		Sometimes		Often		Most of the time	
*Question 1: Physical well-being*								
Over the past month, how often has each of the following interfered with your child’s daily activities?								
Fatigue (having very little energy)	□		□		□		□	
Pain	□		□		□		□	
Poor sleep	□		□		□		□	
Medication (side effects)	□		□		□		□	
*Question 2: Social well-being*	□		□		□		□	
Over the last month, how often has your child’s condition interfere with the things your child likes to do (e.g. playing sport, going to the park, playing out, socialising with friends).								
*Question 3: Emotional well-being*	□		□		□		□	
Over the last month, how often has your child felt sad/worried or frustrated?								

PREM: To be completed after seeing the doctor								
Patient experience		Not at all	A bit		Mostly		Fully	
*Question 1:* During today’s hospital visit, were your/your child’s questions and concerns listened to and answered in a way that you/your child could understand?		□	□		□		□	
*Question 2:* How well do you and your child understand your child’s treatment plan?		□	□		□		□	
*Question 3:* How well supported do you and your child feel in between hospital visits?		□	□		□		□	
*Question 4*: Was the environment in which you waited today suitable for you/your child and your family (those attending the appointment)		□	□		□		□	
Patient experience		No unacceptable delays	Unacceptable delay					
			<15 min	15–30 min	30 min to 1 h	1–2 h		>2 h
*Question 5:* From your appointment time today did you experience a delay you felt unacceptable and if so by how long?		□	□	□	□	□		□

**Table 4:** Continued

Adolescent version of PROM and PREM questionnaires (young person aged >11 years)								
Completed by:		Young person			Other (relationship to child)			
		□			□			
PROM: To be completed prior to seeing the doctor								
Patient outcome		Never	Sometimes	Often	Most of the time			
*Question 1: Physical well-being*								
Over the past month, how often has each of the following interfered with your daily activities?								
Fatigue (having very little energy)		□	□	□	□			
Pain		□	□	□	□			
Poor sleep		□	□	□	□			
Medication (side effects)		□	□	□	□			
*Question 2: Social well-being*		□	□	□	□			
Over the last month, how often has your condition interfered with the things you like to do (e.g. playing sport, going to the park, playing out, socialising with friends)?								
*Question 3: Emotional well-being*		□	□	□	□			
Over the last month, how often have you felt sad/worried or frustrated?								

PREM: To be completed after seeing the doctor								
Patient experience		Not at all	A bit	Mostly	Fully			
*Question 1:* During today’s hospital visit, were your questions and concerns listened to and answered in a way that you could understand?		□	□	□	□			
*Question 2:* How well do you understand your treatment plan?		□	□	□	□			
*Question 3:* How well supported do you feel in between hospital visits?		□	□	□	□			
*Question 4*: Was the environment in which you waited today suitable for you and your family (those attending the appointment)		□	□	□	□			
Patient experience		No unacceptable delays	Unacceptable delay					
			<15 min	15–30 min	30 min to 1 h	1–2 h		>2 h
*Question 5:* From your appointment time today did you experience a delay you felt unacceptable and if so by how long?		□	□	□	□	□		□

PREM, patient and carer reported experience measure; PROM, patient and carer reported outcome measure.

Feedback from the two additional family groups (Scotland and Oxford, April 2016) was very similar. Average time to complete the questionnaire was 3.44 (s.d. 1.49) min for group 1 and 3.45 (s.d. 1.51) min for group 2; 75% and 100% of groups 1 and 2, respectively, reported the questionnaire to be ‘easy’ or ‘very easy’ to complete and 85% and 91%, respectively, were ‘happy’ with the wording used.

### Final list of audit data items and associated data dictionary

The full list of data items (n = 32) plus the PROM/PREM questionnaire (n = 7) is shown in Table 5. The associated data dictionary that would need to be collected to quantify the measures and the outcomes is provided online (supplementary Table S2, available at *Rheumatology* Online).

**Table 5:** Audit data items

Section	Data item
General (dates)	1.1 NHS number of patient (Scotland: CHI number; Northern Ireland: H and C number)
	1.2 Date of attendance
	1.3 Date of referral letter arriving in rheumatology department
	1.4 Date of first appointment offered in a rheumatology clinic
	1.5 Date of first appointment in a rheumatology clinic
	1.6 Date of first eye screen
Demographics	2.1 Date of birth
Diagnosis	3.1 ILAR sub-type
Medication	4.1 Medication name
	4.2 Route
	4.3 Did the decision to treat with steroid injection specify a dedicated paediatric general anaesthesia list?
	4.4 Date of decision to treat or change treatment
	4.5 Date treatment started/date of single treatment
	4.6 Date patient was counselled before starting a DMARD
	4.6 Date patient was counselled before starting a biologic agent
	4.7 Date medication stopped or changed
	4.8 Reason for stopping or changing medication
Clinic organization	5.1 Clinic organization
Research	6.1 Is the patient eligible for the recruitment to the BSPAR Etanercept Cohort Study?
	6.2 Has the patient been recruited to the BSPAR Etanercept Cohort Study?
	6.3 Is the patient eligible for recruitment to the BCRD study?
	6.4 Has the patient been recruited to the BCRD study?
(Core) Outcome variables	7.1.A Active joint assessment
	7.1.B Swollen joint assessment
	7.1.C Tender joint assessment
	7.2 Physician global assessment
	7.3 Patient/parent global assessment of overall well-being
	7.4 CHAQ/HAQ
	7.5.A ESR
	7.5.B Plasma viscosity
	7.6 Date COVs assessed
	7.7 Uveitis status at most recent eye examination

BCRD: Biologics for Children with Rheumatic Diseases; BSPAR: British Society for Paediatric and Adolescent Rheumatology.

## Discussion

This report describes the highly consultative consensus-based methodology underpinning development of a JIA-specific national audit tool including identification of key quality measures for inclusion, and selection of optimal clinician and patient/parent reported outcome tools to understand the impact of variations in service delivery.

National audit programmes aim to improve disease outcome for patients. In the UK, the Royal College of Paediatrics and Child Health supports national audit programmes in paediatric diabetes, neonatal care and epilepsy [25]. Such programmes provide an overview of disease related outcomes and provide potentially powerful benchmarking tools. In diabetes care there is some evidence that investment in regional networks and the introduction of a Best Practice Tariff mandating participation in audit has resulted in some improvements in outcome, but this is still relatively limited and also variable geographically [26]. There are synergies between all chronic diseases of childhood—fundamentally improving the quality of care for children is not only important to control symptoms and alleviate distress, but will also reduce lifetime risk from complications of the disease. A further synergy is with inflammatory arthritis in adulthood. The first national clinical audit for rheumatoid and early inflammatory arthritis highlighted geographically variable gaps between National Institute for Health and Care Excellence standards and existing care [13]. It is reasonable to hypothesize that the same gaps are likely to exist in the provision of paediatric care.

Implementation of a national audit programme for JIA will require a staged approach. The first step will be an initial data collection exercise generating benchmarking data against which robust and validated quality standards can be set. This will facilitate effective quality assessment for NHS England commissioning of specialized services. The second data collection phase will assess how well the standards are being met in terms of the quality, safety, outcome and experience of care for CYP with JIA.

Future participation of all PRh NHS Providers in a national clinical audit process evaluating the care of CYP with JIA will capture a more complete impression of what is happening at local (Trust), regional and national levels. The completion of the audit tool could in itself be considered a quality measure for the delivery of care in JIA. Collecting audit data in itself (challenging though this is) is simply not enough—the data must be utilized to actually deliver the change required to drive quality improvements and this requires concerted effort and commitment from all stakeholders’ services and access to fit-for-purpose IT systems.

Describing and understanding current clinical practice through national clinical audit will enable the development of robust and meaningful quality standards that will be used to drive improvements in clinical care and outcomes for JIA. Important national audit questions and quality measures may change over time. In particular, the current audit tool does not include an assessment of damage. Clinical and radiological evidence of damage occurs relatively late in JIA and damage assessment tools are not currently used routinely in clinical practice. Although a valid and meaningful assessment of damage could not be included in the audit tool at this time, damage may merit inclusion in future iterations, as damage assessment tools evolve further.

The active participation of the wider PRh clinical and research communities across the UK is an important strength of this study. It demonstrates the widespread support for projects informing best practice, the need for greater collaboration between centres, and support for a consensus process to agree and define a feasible dataset (i.e. set of data items to be collected) to encompass the needs of patients and families, clinical teams, research teams, service providers and commissioners. Although we acknowledge that our primary aim of assessing all patients with new onset JIA may be aspirational, the active participation of the wider PRh community in this and other national projects suggests that identification of all new patients could be a realistic expectation.

Patient experience and outcomes are key quality indicators but few condition-specific paediatric PREMs are available. A further strength of the study is the methodologically sound development of novel PROM/PREM tools specific to JIA and developed in consultation with the BSPAR Parents Group.

A major challenge for the PROM/PREM development group was achieving agreement on which broad themes and sub-themes to include. While agreement was achieved, a number of important sub-themes have been excluded. Future development of an electronic data collection tool may enable inclusion of further data items. The PROM/PREM tools require further validation and piloting prior to incorporation into standard clinical practice.

A consensus agreed core dataset for JIA (CAPTURE-JIA), which incorporates the audit data items, has been developed and is currently being piloted. Once finalized, embedding the collection of CAPTURE-JIA data items into routine clinical care would facilitate a national audit programme.

The longer-term goal is development of an electronic data collection tool with routine integration of data collection into electronic patient records. Improved documentation (and auditing) of service delivery at local (trust), regional (clinical networks) and national level against these important clinical and consumer reported outcomes will likely identify potential areas for healthcare quality improvement, key to improving clinical outcomes for this vulnerable group of patients.

Implementation of the proposed national audit tool should facilitate investigation of variability between services and across networks, identify current levels of care and drive a long-term quality improvement programme for children and young people with JIA. Healthcare providers designated as providing paediatric rheumatology services should be held accountable to address inadequacies in care and recommendations when demonstrated using the validated audit tool.

## Supplementary Material

Supplementary Tables

Supplementary Data
