# Supplementary material for: Development of a national audit tool for juvenile idiopathic arthritis: a BSPAR project funded by the Health Care Quality Improvement Partnership
Source: Rheumatology (Oxford). 2017 Oct 23;57(1):140–51. doi: 10.1093/rheumatology/kex322 (PMC5850477; doi:10.1093/rheumatology/kex322)
Supplement: Supplementary Tables [file rhe-17-0241-file002_kex322.docx]

**Development of a national audit tool for juvenile idiopathic arthritis: a BSPAR project funded by the Health Care Quality Improvement Partnership**

**Supplementary Table S1: Committee Structure and Membership**

| Name | Background |
| --- | --- |
| **Scientific Steering Committee** | |
| Gavin Cleary | Consultant Paediatric Rheumatologist (Chair) |
| Flora McErlane | Consultant Paediatric Rheumatologist |
| Kathy Bailey | Consultant Paediatric Rheumatologist |
| Clarissa Pilkington | Consultant Paediatric Rheumatologist |
| Mark Friswell | Consultant Paediatric Rheumatologist |
| Wendy Thomson | Professor of Genetic Epidemiology |
| Gillian Armitt | Project manager |
| Joanna Cobb | Project manager |
| Drew Fell | Clinical Nurse Specialist |
| Helen Strike | Clinical Nurse Specialist |
| Sarah Hartfree | Clinical Nurse Specialist |
| Sharon Douglas | Parent Representative |
| **Expert Review Group** | |
| Gavin Cleary | Consultant Paediatric Rheumatologist |
| Flora McErlane | Consultant Paediatric Rheumatologist |
| Kathy Bailey | Consultant Paediatric Rheumatologist |
| Dan Hawley | Consultant Paediatric Rheumatologist |
| Kate Armon | Consultant Paediatric Rheumatologist |
| Lucy Wedderburn | Professor of Paediatric Rheumatologist |
| Penny Davies | Consultant Paediatric Rheumatologist |
| Karen Davies | Consultant Paediatric Rheumatologist |
| Jeremy Camilleri | Consultant Paediatric Rheumatologist |
| Phil Riley | Consultant Paediatric Rheumatologist |
| Catherine Lees | Consultant Paediatrician |
| Zeba Jamal | Consultant Paediatrician |
| Clarissa Pilkington | Consultant Paediatric Rheumatologist |
| Mark Friswell | Consultant Paediatric Rheumatologist |
| Wendy Thomson | Professor of Genetic Epidemiology |
| Gillian Armitt | Project manager |
| Joanna Cobb | Project manager |
| Drew Fell | Clinical Nurse Specialist |
| Helen Strike | Clinical Nurse Specialist |
| Ellie Potts | Specialist Physiotherapist |
| Sharon Douglas | Parent Representative |
| **PROM/PREM Expert Group** | |
| Wendy Costello | BSPAR Parents Group |
| Terry Cox | BSPAR Parents Group |
| Hayley Dale | BSPAR Parents Group |
| Glenda Dalton | BSPAR Parents Group |
| Sarah Dalton | BSPAR Young Person |
| Sharon Douglas | BSPAR Parents Group |
| Chaya Fekete | BSPAR Parents Group |
| Vicky Green | BSPAR Parents Group |
| Debbie Janson | Clinical studies Group |
| Michael Kent | BSPAR Parents Group |
| Shane Kirrane | BSPAR Parents Group |
| Anne Meredith | BSPAR Parents Group |
| David Parker | BSPAR Parents Group |
| Allan Smith | BSPAR Parents Group |
| Simon Stone | Clinical Studies Group |
| Amy Westcott | BSPAR Young Person |
| Catherine Wright | Clinical Studies Group |

**Supplementary Table S2: Data Dictionary**

(The validation rules ensure data entries are suitable for inclusion in a centralised database)

| \| Data item: \| **1.1 Patient NHS number (Scotland: CHI number; Northern Ireland: H&C number)** \| \| --- \| --- \| \| Section: \| General \| \| Definition: \| NHS number of patient (Scotland: CHI number of patient; Northern Ireland: H&C number of patient) \| \| Format: \| NHS and CHI numbers: numeric; same as HSCIC (n10).  H&C: alphanumeric; n3.n3.n4 \| \| Validation rules: \| H&C: must be within range 320,000,001 to 399,999,999 plus check digit.  CHI: the first digit of the CHI number must be 3 or less. \| \| Options: \| N/A \| \| Collection: \| All patients, first visit and all follow-ups \| \| Related data items: \| None \|  \| Data item: \| **1.2 Date of attendance** \| \| --- \| --- \| \| Section: \| General \| \| Definition: \| Date of the patient's attendance in clinic. Date to be recorded for every consultation between patient / family and any member of the rheumatology MDT. \| \| Format: \| Date DD/MM/YYYY \| \| Validation rules: \| Today's date minus one year *<=* Date of Attendance <= today's date \| \| Options: \| N/A \| \| Collection: \| All patients, first visit and all follow-ups \| \| Related data items: \| Is this the pateints first appointment (Y/N) \|  \| Data item: \| **1.3 Date of referral letter being received in rheumatology department** \| \| --- \| --- \| \| Section: \| General \| \| Definition: \| Date of the first appointment in rheumatology clinic which is offered to new patient. \| \| Format: \| Date DD/MM/YYYY \| \| Validation rules: \| Today's date minus 2 years >=previous audit date <= today's date \| \| Options: \| N/A \| \| Collection: \| All new patients, per audit period \| \| Related data items: \| None \|  \| Data item: \| **1.4 Date of first appointment offered in a rheumatology clinic** \| \| --- \| --- \| \| Section: \| General \| \| Definition: \| Date of the first appointment in rheumatology clinic which is offered to new patient. \| \| Format: \| Date DD/MM/YYYY \| \| Validation rules: \| Today's date minus 2 years >=previous audit date <= today's date \| \| Options: \| N/A \| \| Collection: \| All patients \| \| Related data items: \| None \|  \| Data item: \| **1.5 Date of first appointment in a rheumatology clinic** \| \| --- \| --- \| \| Section: \| General \| \| Definition: \| Date the patient attended their first clinic appointment. \| \| Format: \| Date DD/MM/YYYY \| \| Validation rules: \| Today's date minus 2 years >=previous audit date <= today's date \| \| Options: \| N/A \| \| Collection: \| All patients \| \| Related data items: \| Date of attendence \|  \| Data item: \| **1.6 Date of first eye screen** \| \| --- \| --- \| \| Section: \| General \| \| Definition: \| Date of the first eye screening with opthamologist. \| \| Format: \| Date DD/MM/YYYY \| \| Validation rules: \| Today's date minus 2 years >=previous audit date <= today's date \| \| Options: \| N/A \| \| Collection: \| All patients \| \| Related data items: \| None \|  \| Data item: \| **2.1 Date of birth** \| \| --- \| --- \| \| Section: \| Demographics \| \| Definition: \| Date of birth of the patient \| \| Format: \| Date DD/MM/YYYY \| \| Validation rules: \| Today's date minus 120 years <= Date of Birth <= today's date \| \| Options: \| N/A \| \| Collection: \| All patients \| \| Related data items: \| None \|  \| Data item: \| **2.2 Sex** \| \| --- \| --- \| \| Section: \| Demographics \| \| Definition: \| The gender of a patient, as stated by the patient or the parent / carer \| \| Format: \| Alphanumeric an1 + options text; same as HSCIC \| \| Validation rules: \| One selection from options \| \| Options: \| Same as HSCIC:  1=Male  2=Female  3=Indeterminate (unable to be classified as either male or female)  X=not recorded \| \| Collection: \| All patients \| \| Related data items: \| None \|  \| Data item: \| **3.1 ILAR sub-type** \| \| --- \| --- \| \| Section: \| Diagnosis \| \| Definition: \| Sub-groups of juvenile idiopathic arthritis, categorised according to the International League of Associations for Rheumatology (ILAR) criteria and as assigned by the clinician. \| \| Format: \| Integer max n2 + options text \| \| Validation rules: \| One selection from options \| \| Options: \| 1 = Systemic arthritis  2 = Oligoarthritis, persistent  3 = Oligoarthritis, extended  4 = Polyarthritis, RF Negative  5 = Polyarthritis, RF Positive  6 = Psoriatic arthritis  7 = Enthesitis Related Arthritis  8 = Undifferentiated arthritis  9 = Diagnosis unclear / classification pending  10 = Diagnosis other than JIA (e.g. now lupus)  99 = Not recorded \| \| Collection: \| All patients at first visit; and at follow-up where clinically indicated (if any new clinical changes present) \| \| Related data items: \| None \|   **Medication data items 4.1 – 4.8** will be collected together for each prescribed anti-rheumatic medication, including injected steroids and excluding uveitis medication.   \| Data item: \| **4.1 Medication name** \| \| --- \| --- \| \| Section: \| Medication for JIA inc. Steroids \| \| Definition: \| Generic and associated trade name(s) of anti-rheumatic drugs that the patient has been prescribed and / or is taking regularly.  Includes injected steroids and over the counter anti-rheumatic medication; excludes uveitis medication. \| \| Format: \| Integer max n2 plus options text \| \| Validation rules: \| One selection from options ( to be updated at the start of the audit) \| \| Options: \| The following list of relevant Drug Types and Medication Names requires inclusion of trade names, including for biosimilars.   1. NSAIDs (Non-steroidal Anti-inflammatory Drugs) – [Ibuprofen(Brufen, ibuprofen, Arthrofen, Ebufac, Rimafen, Calprofen, Feverfen, Nurofen, Brufen Retard), diclofenac sodium(voltarol, Diclomax SR, Diclomax Retard, Motifene) , diclofenac potassium (Voltarol Rapid), indomethacin (Indometacin, Indolar SR, Pardelprin), piroxicam (Piroxicam, Feldene), naproxen (Naproxen, Naprosyn), meloxicam (meloxicam), other – please specify] 2. Corticosteroids [Prednisolone (prednisolone, Deltacortril, Pred Forte, Predsol,Minims prednisolone sodium phosphate), methylprednisolone (Medrone, Solu-Medrone, Depomedrone) Triamcinolone hexacetonide (lederlon), Triamcinolone acetonide (Kenalog, Adcortyl), methylprednisolone acetate (Depo-Medrone, Depo-Medrone with Lidocaine), hydrocortisone (Hydrocortisone, Canesten HC, Dioderm, Mildioson, Alphaderm, Canesten HC, Daktacort, Fucidin H, Nystaform-HC, Terra-Cortril, Timodine) other – please specify] 3. DMARDS (Disease Modifying Anti- Rheumatic Drugs)    1. Synthetic DMARDs [includes – methotrexate (methotrexate, Metoject), hydroxychloroquine (Hydroxychloroquine, Quinoric, Plaquenil), sulfasalazine (Sulfasalazine, Salazopyrin), azathioprine (Azathioprine, Imuran), chloroquine (Avloclor, Malarivon, Nivaquine), mycophenolate mofetil (Arzip, cellcept), leflunomide (leflunomide, Arava), cyclophosphamide (Cyclophosphamide), ciclosporin (ciclosporin, capimune, capsorin, Deximiune, Neoral, Sandimun), other – please specify]    2. Biological DMARDs (licensed and unlicensed) [includes – anakinra (Kineret), infliximab (Remicade), biosimilar Infliximab (Remsima, Inflectra) etanercept (Enbrel), adalimumab(Humira), rituximab (MabThera), abatacept (Orencia), tocilizumab (RoActemra), Certolizumab Pegol (Cimzia), other – please specify]. 4. Others \| \| Collection: \| All patients, all visits. \| \| Related data items: \| 1. Drug type (NSAIDS, corticosteroids, synthetic DMARDS, biological DMARDS, others)  2. 'Other – please specify' text fields for corticosteroids and synthetic and biologic DMARDs \|  \| Data item: \| **4.2 Route** \| \| --- \| --- \| \| Section: \| Medication \| \| Definition: \| Route of administration of the prescribed medication, including injected steroids. Not collected for uveitis treatment and over the counter medication. \| \| Format: \| Integer max n1 + options text \| \| Validation rules: \| One selection from options \| \| Options: \| 1=Oral / PO  2=Sub-cutaneous / SC  3=Intra-muscular / IM  4=Intra-venous / IV  5=Intra-articular / IA  6=Intraocular  7=Other (please specify)  8=Not specified \| \| Collection: \| All patients, all visits where a decision to treat with prescribed anti-rheumatic drugs is made or there is a change to an existing treatment. \| \| Related data items: \| 1. 'Other – please specify' text. \|  \| Data item: \| **4.3 Did the decision to treat with steroid injection specify a dedicated Paediatric GA list?** \| \| --- \| --- \| \| Section: \| Medication \| \| Definition: \| Record of whether the decision to treat with steroid injection specified a dedicated paediatric general anaethesia list. \| \| Format: \| Integer max n1 plus options text \| \| Validation rules: \| One selection from options \| \| Options: \| 0=No  1=Yes  2= Not known \| \| Collection: \| All patients receving steroid treatment \| \| Related data items: \| None \|  \| Data item: \| **4.4 Date of decision to treat or change treatment** \| \| --- \| --- \| \| Section: \| Medication for JIA inc. Steroids \| \| Definition: \| Date of decision to treat with prescribed anti-rheumatic medication, or decision to change the dose and / or frequency and / or route of existing anti-rheumatic medication.  Includes injected steroids but excludes uveitis treatment and over the counter medication. \| \| Format: \| Date DD/MM/YYYY. Where day and / or month is unknown, put '01' in the relevant field(s); if year is unknown, do not complete this data item. \| \| Validation rules: \| Date of Diagnosis <= Date of Decision to Treat or Change Treatment <= today's date \| \| Options: \| N/A \| \| Collection: \| All patients, all visits where a decision to treat with prescribed anti-rheumatic drugs is made. \| \| Related data items: \| 1. Date treatment started \|  \| Data item: \| **5.1 Clinic type** \| \| --- \| --- \| \| Section: \| Clinic organistation \| \| Definition: \| Type of clinical appointment. \| \| Format: \| Integer max n1 plus options text \| \| Validation rules: \| One selection from options \| \| Options: \| 1= Specialist paediatric rheumatology clinic in designated specialist centre  2= Adolescent paediatric rheumatology clinic with paediatric rheumatologist  3= Adolescent paediatric rheumatology clinic with adult rheumatologist  4= Dedicated paediatric rheumatology clinic in secondary care/network centre. Clinical lead: paediatric rheumatologist  5= Dedicated paediatric rheumatology clinic in secondary care/network centre. Clinica Lead: adult rheumatologist with special interest  6= Dedicated paediatric rheumatology clinic in secondary care/network centre. Clinical lead: paediatrician with special interest  7= General paediatric clinic (with mixed patients)  8= Adult rheumatology clinic (with mixed patients)  9= Other clinic type  0= Not known \| \| Collection: \| All patients, each clinic visit \| \| Related data items: \| None \|  \| Data item: \| **6.1 Is the patient eligible for recruitment to the BSPAR Etanercept Cohort Study?** \| \| --- \| --- \| \| Section: \| Research \| \| Definition: \| Record of whether the patient is eligible for recruitment to the BSPAR Etanercept Cohort Study.  Eligiblity for the biologics arm includes:   - Diagnosis of JIA - Age under 18 - Informed consent within six months of the first ever dose of etanercept - Can have received previous biologics   Eligibility for the methotrexate arm includes:   - Diagnosis of JIA - Age under 18 - Informed consent within six months of the first ever dose of methotrexate - Must be biologic naive \| \| Format: \| Integer max n1 plus options text \| \| Validation rules: \| One selection from options \| \| Options: \| 0=No  1=Yes  2= Not known \| \| Collection: \| All patients, each clinic visit until eligible \| \| Related data items: \| None \|  \| Data item: \| **6.2 Has the patient been recruited to the BSPAR Etanercept Cohort Study?** \| \| --- \| --- \| \| Section: \| Research \| \| Definition: \| Record of whether the patient has been recruited to the BSPAR Etanercept Cohort Study. \| \| Format: \| Integer max n1 plus options text \| \| Validation rules: \| One selection from options \| \| Options: \| 0=No  1=Yes  2= Information provided  3=Declined  4= Not known \| \| Collection: \| All patients, each clinic visit until recruited or refused \| \| Related data items: \| None \|  \| Data item: \| **6.3 Is the patient eligible for recruitment to the BSPAR BCRD Cohort Study?** \| \| --- \| --- \| \| Section: \| Research \| \| Definition: \| Record of whether the patient is eligible for recruitment to the BSPAR Biologics for Children with Rheumatic Diseases (BCRD) Cohort Study.  Eligiblity for the biologics arm includes:   - Diagnosis of JIA - Age under 18 - Informed consent within six months of the first ever dose of the registration drug - Can have received previous biologics (including previous exposure to etanercept) \| \| Format: \| Integer max n1 plus options text \| \| Validation rules: \| One selection from options \| \| Options: \| 0=No  1=Yes  2= Not known \| \| Collection: \| All patients, each clinic visit until eligible \| \| Related data items: \| None \| \| Data item: \| **6.4 Has the patient been recruited to the BSPAR BCRD Cohort Study?** \| \| Section: \| Research \| \| Definition: \| Record of whether the patient has been recruited to the BSPAR Biologics for Children with Rheumatic Diseases (BCRD) Cohort Study. \| \| Format: \| Integer max n1 plus options text \| \| Validation rules: \| One selection from options \| \| Options: \| 0=No  1=Yes  2= Information provided  3=Declined  4= Not known \| \| Collection: \| All patients, each clinic visit until recruited or refused \| \| Related data items: \| None \|  \| Data item: \| **7.1A Active joint assessment** \| \| --- \| --- \| \| Section: \| (Core) Outcome Variables \| \| Definition: \| List of joints with active arthritis, defined as the presence of swelling not due to currently inactive synovitis or to bony enlargement; or if no swelling is present joints with limitation of motion accompanied by heat, pain, or tenderness.  Either the Active Joint Assessment OR both the Swollen and Tender Joint Assessments should be collected. Active joints can be determined from the Swollen and Tender Joint Assessments. \| \| Format: \| Result for each joint: integer max n1 + options text \| \| Validation rules: \| For each joint, one selection from options \| \| Options: \| Named joints Y / N (mannequin preferred)  In database: N=0; Y=1 \| \| Collection: \| All patients, first visit and all follow-ups \| \| Related data items: \| 1. Table of joints (format: integer max n2 + joint name) *(to support different joint mannequins / tables in CAPTURE-JIA)*  2. No active joints (blank / tick)  23. (Calculation: number of active joints) \|  \| Data item: \| **7.1B Swollen joint assessment** \| \| --- \| --- \| \| Section: \| (Core) Outcome Variables \| \| Definition: \| List of swollen joints.  Either the Active Joint Assessment OR both the Swollen and Tender Joint Assessments should be collected. Active joints can be determined from the Swollen and Tender Joint Assessments. \| \| Format: \| Result for each joint: integer max n1 + options text \| \| Validation rules: \| For each joint, one selection from options \| \| Options: \| Named joints Y / N (mannequin preferred)  In database: N=0; Y=1 \| \| Collection: \| All patients, first visit and all follow-ups \| \| Related data items: \| 1. Table of joints (format: integer max n2 + joint name) *(to support different joint mannequins / tables in CAPTURE-JIA)*  2. No swollen joints (blank / tick)  3. (Calculation: number of active joints) \|  \| Data item: \| **7.1C Tender joint assessment** \| \| --- \| --- \| \| Section: \| (Core) Outcome Variables \| \| Definition: \| List of tender joints, defined as the presence of pain at rest with pressure or on movement of the joint.  Either the Active Joint Assessment OR both the Swollen and Tender Joint Assessments should be collected. Active joints can be determined from the Swollen and Tender Joint Assessments. \| \| Format: \| Result for each joint: integer max n1 + options text \| \| Validation rules: \| For each joint, one selection from options \| \| Options: \| Named joints Y / N (mannequin preferred)  In database: N=0; Y=1 \| \| Collection: \| All patients, first visit and all follow-ups \| \| Related data items: \| 1. Table of joints (format: integer max n2 + joint name) *(to support different joint mannequins / tables in CAPTURE-JIA)*  2. No tender joints (blank / tick)  3. (Calculation: number of active joints) \|  \| Data item: \| **7.2 Physician Global Assessment** \| \| --- \| --- \| \| Section: \| (Core) Outcome Variables \| \| Definition: \| Physician global assessment of overall disease activity \| \| Format: \| Numeric; max n2.n1 \| \| Validation rules: \| Must be in range 0.0 - 10.0 cm \| \| Options: \| 0.0 - 10.0 cm VAS. Anchors: 0.0 =Very well; 10.0cm=Very unwell \| \| Collection: \| All patients, first visit and all follow-ups \| \| Related data items: \| None \|  \| Data item: \| **7.3 Patient / Parent Global Assessment of Overall Well-Being** \| \| --- \| --- \| \| Section: \| (Core) Outcome Variables \| \| Definition: \| Patient or parent / carer global assessment of patient well-being \| \| Format: \| Numeric; max n2.n1 \| \| Validation rules: \| Must be in range 0.0 - 10.0 cm \| \| Options: \| 0.0 - 10.0 cm VAS. Anchors: 0.0 =Very well; 10.0cm=Very unwell \| \| Collection: \| All patients, first visit and all follow-ups \| \| Related data items: \| 1. Was the Patient / Parent Global Assessment completed by the patient or the parent / carer?  2. If completed by parent / carer, which one? (options include mother, father, grandparent, other) \|  \| Data item: \| **7.4 CHAQ / HAQ** \| \| --- \| --- \| \| Section: \| (Core) Outcome Variables \| \| Definition: \| Calculated result of the Childhood Health Assessment Questionnaire (CHAQ) or Health Assessment Questionnaire (HAQ). Either CHAQ or HAQ should be completed, as appropriate to age. \| \| Format: \| Numeric; max n1.n3 \| \| Validation rules: \| Must be in range 0.000 - 3.000; allowed values are only 0.125, 0.250, etc (in increments of 0.125) \| \| Options: \| N/A \| \| Collection: \| All patients, first visit and all follow-ups \| \| Related data items: \| 1. Was the CHAQ or HAQ completed?  2. Was the CHAQ / HAQ completed by the patient or the parent / carer? (default to ‘Patient’ for HAQ)  3. If completed by parent / carer, which one? (options include mother, father, grandparent, other) \|  \| Data item: \| **7.5.A ESR** \| \| --- \| --- \| \| Section: \| (Core) Outcome Variables \| \| Definition: \| Blood test measuring erythrocyte sedimentation rate. If ESR testing is not available, plasma viscosity should be captured; however, ESR is recommended as it is a Core Outcome Variable. \| \| Format: \| Numeric; max n3 \| \| Validation rules: \| Must be in range 0 - 200 mm/hr \| \| Options: \| Integer value in mm/hr \| \| Collection: \| All patients, first visit and follow-ups where clinically indicated. Should be collected within two weeks of the Date of Attendance at which other Core Outcome Variables are assessed. \| \| Related data items: \| 1. Not tested? (blank / Y)  2. Date COVs assessed (date of assessment of ESR) \|  \| Data item: \| **7.5.B Plasma Viscosity** \| \| --- \| --- \| \| Section: \| (Core) Outcome Variables \| \| Definition: \| Blood test measuring plasma viscosity. To be captured instead of ESR if ESR testing is not available; however, ESR is recommended as it is a Core Outcome Variable. \| \| Format: \| Numeric; n1.n2 \| \| Validation rules: \| Must be in range 1.00 - 2.00 centipoise (cp) \| \| Options: \| Numeric value \| \| Collection: \| First visit and follow-ups where clinically indicated, for all patients. Should be collected within two weeks of the Date of Attendance at which Core Outcome Variables are assessed. \| \| Related data items: \| 1. Not tested? (blank / Y)  2. Date of assessment of Plasma Viscosity \| \| Note: \| Added as an alternative to ESR following discussion during the voting round. \|  \| Data item: \| **7.6 Date COVs assessed** \| \| --- \| --- \| \| Section: \| (Core) Outcome Variables \| \| Definition: \| For each Core Outcome Variable* (*Active Joint Count, Limited Joint Count, ESR or CRP, CHAQ / HAQ, Physician Global Assessment, Patient / Parent Global Assessment of Overall Well-Being), the date it was assessed, if different from the Date of Attendance. \| \| Format: \| Date DD/MM/YYYY \| \| Validation rules: \| Today's date minus one year <= Date COV assessed < today's date \| \| Options: \| N/A \| \| Collection: \| All patients, all visits where COVs are assessed, or have been assessed since the last visit. \| \| Related data items: \| 1. All the following data items: Active Joint Count, Limited Joint Count, ESR or CRP, CHAQ / HAQ, Physician Global Assessment, Patient / Parent Global Assessment of Overall Well-Being \|  \| Data item: \| **7.7 Uveitis history** \| \| --- \| --- \| \| Section: \| (Core) Outcome Variables \| \| Definition: \| History of uveitis (all types) \| \| Format: \| In database, for each option, integer n1 \| \| Validation rules: \| If 'History of uveitis (any type, ever)' = N or Don't know, no other uveitis-related options are seen.  Database: 0 = N, 1 = Y, 9 = Don't know \| \| Options: \| Question 1: History of uveitis (any type, ever)? Y / N / Don't know  Question 2: Acute anterior uveitis (ever) Y / N / Don't know  Question 3: Chronic anterior uveitis (ever) Y / N / Don't know  Question 4: Other types of uveitis (ever) Y / N / Don’t know \| \| Collection: \| All visits, all patients. Patients should be referred for screening on their first visit. \| \| Related data items: \| 1. Uveitis status at most recent eye examination  2. If options Q1-4 is Y, additoinal question: one or both eyes? \| \| Note: \| Handling of uveitis split into history and current status at the World Café stage of the workshop. \| |
| --- | --- | --- | --- | --- | --- | --- | --- | --- | --- | --- | --- | --- | --- | --- | --- | --- | --- | --- | --- | --- | --- | --- | --- | --- | --- | --- | --- | --- | --- | --- | --- | --- | --- | --- | --- | --- | --- | --- | --- | --- | --- | --- | --- | --- | --- | --- | --- | --- | --- | --- | --- | --- | --- | --- | --- | --- | --- | --- | --- | --- | --- | --- | --- | --- | --- | --- | --- | --- | --- | --- | --- | --- | --- | --- | --- | --- | --- | --- | --- | --- | --- | --- | --- | --- | --- | --- | --- | --- | --- | --- | --- | --- | --- | --- | --- | --- | --- | --- | --- | --- | --- | --- | --- | --- | --- | --- | --- | --- | --- | --- | --- | --- | --- | --- | --- | --- | --- | --- | --- | --- | --- | --- | --- | --- | --- | --- | --- | --- | --- | --- | --- | --- | --- | --- | --- | --- | --- | --- | --- | --- | --- | --- | --- | --- | --- | --- | --- | --- | --- | --- | --- | --- | --- | --- | --- | --- | --- | --- | --- | --- | --- | --- | --- | --- | --- | --- | --- | --- | --- | --- | --- | --- | --- | --- | --- | --- | --- | --- | --- | --- | --- | --- | --- | --- | --- | --- | --- | --- | --- | --- | --- | --- | --- | --- | --- | --- | --- | --- | --- | --- | --- | --- | --- | --- | --- | --- | --- | --- | --- | --- | --- | --- | --- | --- | --- | --- | --- | --- | --- | --- | --- | --- | --- | --- | --- | --- | --- | --- | --- | --- | --- | --- | --- | --- | --- | --- | --- | --- | --- | --- | --- | --- | --- | --- | --- | --- | --- | --- | --- | --- | --- | --- | --- | --- | --- | --- | --- | --- | --- | --- | --- | --- | --- | --- | --- | --- | --- | --- | --- | --- | --- | --- | --- | --- | --- | --- | --- | --- | --- | --- | --- | --- | --- | --- | --- | --- | --- | --- | --- | --- | --- | --- | --- | --- | --- | --- | --- | --- | --- | --- | --- | --- | --- | --- | --- | --- | --- | --- | --- | --- | --- | --- | --- | --- | --- | --- | --- | --- | --- | --- | --- | --- | --- | --- | --- | --- | --- | --- | --- | --- | --- | --- | --- | --- | --- | --- | --- | --- | --- | --- | --- | --- | --- | --- | --- | --- | --- | --- | --- | --- | --- | --- | --- | --- | --- | --- | --- | --- | --- | --- | --- | --- | --- | --- | --- | --- | --- | --- | --- | --- | --- | --- | --- | --- | --- | --- | --- | --- | --- | --- | --- | --- | --- | --- | --- | --- | --- | --- | --- | --- | --- | --- | --- | --- | --- | --- | --- | --- | --- | --- | --- | --- | --- | --- | --- | --- | --- | --- | --- | --- | --- | --- | --- | --- | --- | --- | --- | --- | --- | --- | --- | --- | --- | --- | --- | --- | --- | --- | --- | --- | --- | --- | --- | --- | --- | --- | --- | --- | --- | --- | --- | --- | --- | --- | --- | --- | --- | --- | --- | --- | --- | --- |
