# Supplementary material for: Development of a national audit tool for juvenile idiopathic arthritis: a BSPAR project funded by the Health Care Quality Improvement Partnership
Source: Rheumatology (Oxford). 2017 Oct 23;57(1):140–51. doi: 10.1093/rheumatology/kex322 (PMC5850477; doi:10.1093/rheumatology/kex322)
Supplement: Supplementary Data [file rhe-17-0241-file004_kex322.docx]

Thank you for reviewing the paper and for including your suggestions and comments. We have responded to each suggestion as follows:

Reviewer: 1
The authors present work related to their ongoing efforts to optimize care for children and adolescents with juvenile idiopathic arthritis (JIA). The authors provide a good rationale for the need for an audit tool and the overall design/aim of the tool. Parts of the manuscript come across more as a policy statement than as a scientific paper, with reference to need to comply with standards, needs.

It would be helpful if they delineated the needs more clearly rather than only referencing the organization that is specifying the needs.

*Thank you for this comment. We have further summarised the need for national clinical audit in this patient group in the introductory paragraphs.*

In addition, the details for how they developed this tool are limited. There is not enough information provided to evaluate how well the study groups reflect UK paediatric rheumatology practice, or children or adolescents with JIA priorities.

*Thank you for this comment. We have included some more detail on the selection and composition of the study groups.*

More details are needed especially in the methods section.
1. committees: while the names and titles/roles of the members are listed, no information was provided as to 
a. how each of these groups were generated;

*This information has been added to the methods (‘development of governance structure’)*

i. were physicians and other health professionals self-nominated, voted upon by a larger membership, etc.

*Representatives from all NHS England Paediatric Rheumatology providers and Paedaitric Rheumatology representatives from Scotland and Wales were invited to participate.A purposeful sample was selected, including representation from >80% all UK sites. This information has been added to the methods section.*

ii. were parents and patients identified by physicians, self-selected, or nominated by larger parent/patient group?

*Patients/parents were identified by the BSPAR parents group, a national consumer group.*

b. how many different sites do the physicians represent, and what percentage of the total UK sites and paediatric rheumatologists does this represent?

*>80% UK PRh NHS providers were represented and this has been added to the methods section.*

c. How many different sites/locations do the patients and parents represent, how many in the different age groups (child vs adolescent), how many different families are involved? What disease subtypes and severity levels/issues are represented by the patients/parents?

*Membership of the PROM/PREM Development Group included patients and parents from England, Scotland and Northern Ireland. To avoid compromising patient confidentiality, we have included limited information regarding geographical location and disease severity.*

2. Consensus: the authors talk about reaching consensus but do not specify what level of agreement they agree to use, and why they chose that level of agreement
a. Page 9: Consensus for primary disease activity measures

*Consensus was taken as agreement of >/=70% to align our work with consensus definitions favoured by the Outcome Measures in Rheumatology (OMERACT) collaboration and the Core Outcome Measures in Effectiveness trials (COMET) initiative; the consensus cut-off of 70% has been used in a number of previous paediatric rheumatology studies. This informarion has been added to the methods.*

3. Audit of 14 UK pediatric rheumatology providers to assess documentation of disease activity measures: page 11
a. how were these 14 selected? How many centers do they represent? How much do they overlap/differ from the steering committee or expert group committees?

*We have added some more detail about the audit methodology and results.*

4. Composition of 2 additional groups of patients and parents that completed PROM and PREM questionnaires
a. How many different families did they represent?
b. How diverse a spectrum of JIA subtypes and severity do they represent?

*We have added some more detailed information regarding the two family groups, again taking care to protect patient confidentiality.*

Another issue is the PROM/PREM development group not following through on their agreed upon level for including themes in the audit. On Page 13, authors report that none of the identified themes/sub-themes met this 73% agreement level. Yet rather than try to work through differences to achieve their standard for agreement, authors report that the group still went ahead in developing questions for some of the themes. No information is provided on how they decided which themes/sub-themes to develop and this deviation raises concerns about the validity of their process. While face validity was found when tested with parent groups, the test groups were small and no information was given as to spectrum of disease subtype and severity represented by these small groups. 

*We have clarified the process through which the group achieved consensus.*

*The broad themes and sub-themes have been added as Table 3.*

*We have added as much information about the composition of the small groups as patient confidentiality allows.*

Reviewer: 2

Comments to be transmitted to the Author The development process of a national audit tool for children and adolescents with JIA is introduced in the paper. The authors and the involved group did a great job. The paper is well written. I have only some comments.
1. PROM and PREMS
a. Was the initial workshop moderated? I assume that the framework of the workshop was an open exchange of experiences between the participants. Who brought together the key messages and operationalized these key points?

*Thank you for this point. I have added some text explaining that the workshop was moderated by a paed rheum consultant with key messages brought together by one of the consumer members of the group.*

b. How did the participants achieve consensus in the questionnaire development workshop in case of disagreement?

*We have clarified this process in the text.*

c. Did you use a rating scale or a simple yes/no question in the online prioritization step?

Simple yes/no (and this has been added to the text).

2. How realistic is the primary aim to assess all patients with new onset JIA?

*It was felt by the SSC and expert group that this is a realistic expectation. All new patients should be seen by a paediatric rheumatology provider. The incidence is 1:10 000 so the workload will not be overly onerous. We have included some reflection on this point in the discussion.*

3. There was a remarkable variability in the collection and documentation of disease activity. Please provide some more detail, what was the main source of variability and disagreement?

*We have added some more detail about the audit methodology and results.*

4. Main results about agreement rates between the participating stakeholders should be reported for the steps including consensus meetings, e.g. the clinical outcome measures and PROMs.

*Thank you. We have added further information accordingly.*

5. The reader of the paper may be interested in some more details about the “important” excluded subthemes and reasons for exclusion.

*We have added the broad themes and subthemes as Table 3.*
